# Women’s decision-making processes and the influences on their mode of birth following a previous caesarean section in Taiwan: a qualitative study

**DOI:** 10.1186/s12884-018-1661-0

**Published:** 2018-01-17

**Authors:** Shu-Wen Chen, Alison M. Hutchinson, Cate Nagle, Tracey K. Bucknall

**Affiliations:** 1 Deakin University, Faculty of Health, School of Nursing and Midwifery, Geelong, Australia; 2 National Taipei University of Nursing and Health Science, School of Nursing, Taipei, Taiwan; 3 Centre for Quality and Patient Safety Research-Monash Health Partnership, Clayton, Australia; 4 James Cook University & Townsville Hospital and Health Service, School of Nursing and Midwifery, Townsville, Australia; 5 Centre for Quality and Patient Safety Research- Alfred Health Partnership, Melbourne, Australia

**Keywords:** Vaginal birth after caesarean (VBAC), Repeat caesarean section (RCS), Mode of birth, Decision-making, Qualitative research, Risk evaluation

## Abstract

**Background:**

Vaginal birth after caesarean (VBAC) is an alternative option for women who have had a previous caesarean section (CS); however, uptake is limited because of concern about the risks of uterine rupture. The aim of this study was to explore women’s decision-making processes and the influences on their mode of birth following a previous CS.

**Methods:**

A qualitative approach was used. The research comprised three stages. Stage I consisted of naturalistic observation at 33-34 weeks’ gestation. Stage II involved interviews with pregnant women at 35-37 weeks’ gestation. Stage III consisted of interviews with the same women who were interviewed postnatally, 1 month after birth. The research was conducted in a private medical centre in northern Taiwan. Using a purposive sampling, 21 women and 9 obstetricians were recruited. Data collection involved in-depth interviews, observation and field notes. Constant comparative analysis was employed for data analysis.

**Results:**

Ensuring the safety of mother and baby was the focus of women’s decisions. Women’s decisions-making influences included previous birth experience, concern about the risks of vaginal birth, evaluation of mode of birth, current pregnancy situation, information resources and health insurance. In communicating with obstetricians, some women complied with obstetricians’ recommendations for repeat caesarean section (RCS) without being informed of alternatives. Others used four step decision-making processes that included searching for information, listening to obstetricians’ professional judgement, evaluating alternatives, and making a decision regarding mode of birth. After birth, women reflected on their decisions in three aspects: reflection on birth choices; reflection on factors influencing decisions; and reflection on outcomes of decisions.

**Conclusions:**

The health and wellbeing of mother and baby were the major concerns for women. In response to the decision-making influences, women’s interactions with obstetricians regarding birth choices varied from passive decision-making to shared decision-making. All women have the right to be informed of alternative birthing options. Routine provision of explanations by obstetricians regarding risks associated with alternative birth options, in addition to financial coverage for RCS from National Health Insurance, would assist women’s decision-making. Establishment of a website to provide women with reliable information about birthing options may also assist women’s decision-making.

**Electronic supplementary material:**

The online version of this article (10.1186/s12884-018-1661-0) contains supplementary material, which is available to authorized users.

## Background

Previous caesarean sections (CS) account for a significant proportion of the high rates of repeat caesarean section (RCS) reported in high income countries [1–3]. While vaginal birth after caesarean (VBAC) is a safe intervention [4], there has been a dramatic decline in VBAC because of concerns about uterine rupture and perinatal death [5]. This is despite evidence that the rates of uterine rupture and perinatal death are relatively low [4]. According to findings from a systematic review of 203 studies, once a woman has experienced a CS, she has a baseline risk for uterine rupture in subsequent pregnancies that is estimated at 3 per 1000 [4]. For women choosing VBAC in a subsequent pregnancy, this baseline risk increases to 4.7 per 1000, compared with 0.3 per 1000 for women choosing RCS. Of concern, however, is that RCS is associated with an increased risk of adverse maternal and neonatal outcomes [6], and has a major economic impact on health care [7].

Several factors influence women’s decision-making regarding birth mode. Historically, it has been reported that, associated with the notion of “once a caesarean, always a caesarean”, obstetricians recommended RCS to women who had had a previous CS [8]. Higher preference for CS has been reported in women with previous CS [9]. While perceptions of safety were a common reason for caesarean section by maternal request (CSMR) without medical indication [10], evidence demonstrates inconsistent findings in maternal and neonatal risks [11]. Caesarean sections by maternal request (CSMR) were associated with higher rates of infection and length of hospital stay and neonatal respiratory morbidity, compared to planned vaginal birth [11]. Knowledge about birth options influences a woman’s ability to make decisions regarding mode of birth. In a survey to explore how information women received in pregnancy affected their childbirth preferences, all (*n* = 34) women who chose a VBAC felt involved in the decision-making, while almost 20% (*n* = 28) of women who underwent a RCS reported not being involved in decision-making [12]. Women also use relevant information from previous birthing experiences to inform a birth decision [13]. According to findings of a qualitative study, 13 Australian women who had a VBAC reported that their previous caesarean experience was unacceptable to them and resulted in an unexpected extended recovery. These experiences reinforced their desire for a subsequent vaginal birth [14]. Fear of birth was related to previous negative birth experience [15]. Additionally, social context has been found to increase women’s fears of birth, such as ‘fear of the unknown’, ‘horror stories’ and ‘general fear for the well-being of the baby’ [16]. In some countries, hospitals restrict access to VBAC based on the capabilities of the service and limitations of VBAC guidelines [17, 18].

Taiwan has high RCS rates [19]. In 2015, a total of 216,229 babies were born in Taiwan; 35.67% (*n* = 77,144) of babies were born by CS; that is, more than one in three women delivered by CS. Of all births, RCS accounted for 14.16% (*n* = 30,622) [19]. High RCS rates are associated with extremely low rates of practicing midwives in Taiwan. In 2016, of all births, 99.89% (*n* = 215,983) of babies were delivered by obstetricians while 0.06% (*n* = 126) of babies were born with midwives’ assistance [19]. Between 1951 and 1971, a midwife was the main health professional to assist women during birth. With cultural vicissitudes and the concomitant rise of medicalisation within health services, numerous organisations adopted obstetrician-provided maternal care in Taiwan [20], similar to North America. Obstetricians are the primary providers of prenatal care for most childbearing women. An obstetrician is present for the birth, and nurses provide intrapartum and postnatal care [21]. As a result of the high CS rates, the Taiwan government reconsidered the role of midwives [22]. Midwifery education was recommenced and the midwifery qualification was advanced to undergraduate level in 1996 and then to a graduate level in 2000 [22].

Previous CS is ranked as the top reason for RCS in Taiwan [23]. While increasing VBAC is an efficient way to lower CS rates in Taiwan [24], the prevalence of VBAC was less than 0.37% (*n* = 806) in 2016 [19]. Although studies from high income countries have indicated that women’s choice of RCS was related to: individual preferences [9]; safety [25]; insufficient information about various modes of birth [12–14]; health professionals’ advice [26] and limitations of guideline and policy [18], these findings may not explain the phenomenon of extremely low VBAC rates in Taiwan. In particular, Taiwan has a different social context from high income countries. Studies conducted in high income countries have focussed on either health professionals’ views or women’s perspectives, but little is known about how cultural context and practice patterns influence women’s decisions. More importantly, a number of quantitative studies have been conducted to examine factors related to CS in Taiwan [27–30]. However, qualitative research to explore decision-making regarding mode of birth in Taiwanese women who have had a previous CS, has not been undertaken. To redress this gap, the present study aimed to explore Taiwanese women’s decision-making processes and the influences on their mode of birth following a previous CS.

## Methods

### Study design

Using a qualitative approach, the research encompassed three stages. Stage 1 consisted of naturalistic observation of obstetric consultations to understand how obstetricians assisted women to make their birth choices. Stage 2 involved interviews with pregnant women to explore their perceptions of the influences on their preferences for mode of birth. Stage 3 consisted of interviews in the postnatal period with the same women who were interviewed in Stage 2. The purpose of the Stage 3 interviews was to capture women’s reflections about the influences on their decisions regarding mode of birth, and the relationship between their decisions and the actual birth mode outcome.

### Setting and participants

The study was conducted in a private, tertiary teaching medical centre in northern Taiwan. At the hospital, there were between 350 and 450 births per month and the CS rate varied between 34% and 38%, consistent with Taiwan’s overall CS rates [19]. A purposive sampling approach was used in this study. Pregnant women who had undergone a previous CS were eligible to be included. Inclusion criteria were: women who were aged 18-45 years of age, fluent in Mandarin or English, 30-32 weeks’ gestation, and had experienced a previous CS. Exclusion criteria included women with a multiple pregnancy, a previous classic CS or myomectomy, and/or high-risk pregnancies (for example, women who had risk factors such as threatened premature labour, hypertension, heart disease, diabetes, epilepsy, or another pre-existing medical problem).

### Data collection and procedures

Data collection included observation, in-depth interviews and field notes. Non-participant observation (the complete observer) was used in order to avoid influencing participants’ decisions. The aim of observation is to seek detailed knowledge of the multiple dimensions of life within the natural setting and to understand participants’ taken-for-granted meanings and rules from the perspective of those being observed [31, 32]. Instead of covert observation, where participants are unaware they are being observed, overt observation was used to study naturally occurring behaviour of participants [31]. Prior to observation, written consent from the obstetricians for the observation was obtained. Each woman participant was invited to participate and informed about how they would be observed and the purpose behind the observation. Interested participants provided written consent for observation of the consultation with the obstetrician.

Interviews were the main method for data collection. Interviews seek to describe the meaning of central themes in the life of the subjects. The main task in interviewing is to understand the meaning of the interviewees [33]. Interviews are particularly useful for obtaining the story behind a participant’s experiences. The interviewer pursues in-depth information around the topic [34]. Two interviews of women were conducted to elicit their perspectives, preferences regarding birth choice before and birth reflections afterwards. A semi-structured interview guide was used for the interview to cover key issues for women participants.

Ethics approval was obtained from the university and hospital Human Research Ethics Committee. Prior to commencement, participants gave written informed consent for participation and the audio-recording of the interviews. The researcher invited eligible women to participate in the study when they attended the registration counter for their prenatal examination at the 33-34 weeks’ gestation visit in the Outpatient Department of Obstetrics and Gynaecology. Interactions between the consulting obstetrician and the pregnant woman were observed and field notes were recorded.

**Fig. 1:**
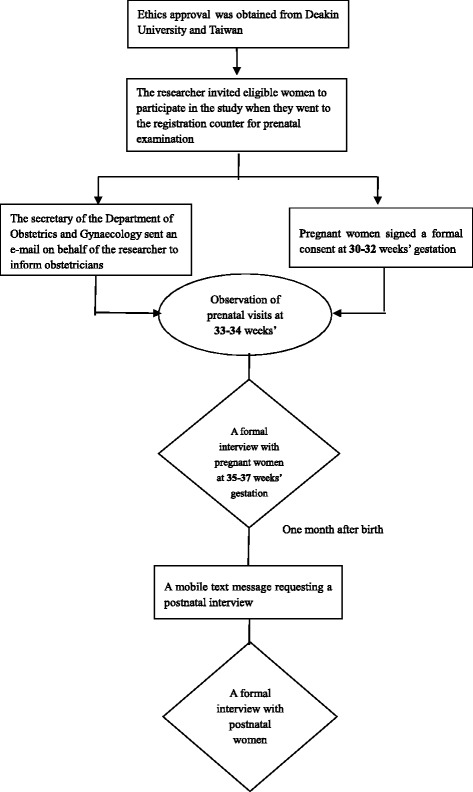
Flow diagram of the recruitment procedure

A prenatal interview with the woman was scheduled to coincide with the woman’s next visit to the obstetrician. The formal face-to-face interview was held at 35-37 weeks’ gestation when the women visited their obstetrician. The quiet waiting room of the Outpatient Department of Obstetrics and Gynaecology was used to conduct the interview while the women waited for their obstetrician appointment. Interviews commenced with a key question, “Could you tell me what is your birth plan regarding mode of birth?” (Additional file 1) Following the provision (before the birth of the child) of signed consent for the postnatal interview, and at approximately 1 month after birth, a mobile text message requesting a postnatal interview was issued to the women to confirm their intention to participate in the interview. The face-to-face interviews were conducted in the waiting room of the Outpatient Department of Obstetrics and Gynaecology or the Neonatal Department after the postnatal women attended their routine follow-up postnatal appointment. The key opening question, “Could you tell me what influenced your decision about mode of birth?” (Additional file 1) guided the conversation. The procedure and recruitment process is illustrated (Fig. 1).

Interviews were transcribed verbatim in Chinese from the digital audio-recording as soon as was practicable after the interview. Once the interviews were transcribed, the researcher verified the transcripts by listening to the recording repeatedly. Two bilingual teachers who were Chinese and had masters’ degrees in Taiwan assisted the researcher to translate interviews from Chinese to English.

### Data analysis

The constant comparative analytic method of grounded theory informed data analysis [35, 36]. Data gathering, analysis and construction proceeded concurrently and coding and memo writing started soon after collection of the first interview and field notes [37]. N-Vivo 10 software package was used for data coding and retrieval, organization and presentation. The four criteria of credibility, confirmability, dependability and transferability were employed to evaluate the trustworthiness of this qualitative research [38]. To ensure credibility, multiple strategies were used: open-ended interviews; field notes; observation and demographic information to build triangulation. The principal researcher who collected data specialised in teaching obstetric nursing, and has worked with students in delivery rooms for over 10 years. The prolonged engagement and extensive experience working with this population was important to establishing credibility with potential participants. Triangulation strategies were also used to reduce the effect of research bias to establish confirmability, including reflexive field notes and observation. Raw data, data analysis products and data reconstruction products were provided to the research team for verification. To ensure dependability, naturalistic observation and individual interview data were compared to examine the consistency of data (cross-validating data). Additionally, the research team discussed and came to consensus on the categories and theming during the stages of analysis. To ensure transferability, the study provides clear and detailed description of the data collection procedure and the process of analysis, as well as the findings. This detailed description will provide other researchers with the opportunity to assess the relevance to their own setting of the knowledge that is generated.

## Results

A total of 24 pregnant women agreed to participate in the study and provided signed consent. Three women were excluded from the study including two women who transferred to another hospital located in southern Taiwan and one woman who miscarried before the prenatal interview. In total, nine obstetricians and 21 pregnant women participated in the study. Women completed a survey to provide their demographic characteristics (Table 1).

**Table 1:** Demographic characteristics of women (*N* = 21)

Characteristics	Number	Percent
Age group (years)		
26-30	3	14.3
31-35	8	38.1
36-40	7	33.3
41-45	3	14.3
Nationality		
Taiwanese	20	95.2
Chinese	1	4.8
Education (highest level achieved)		
High school	11	52.3
College	2	9.5
Undergraduate	6	28.6
Postgraduate	2	9.5
Professional		
Business	4	19.0
Health	3	14.3
Housewife	5	23.8
Other	9	42.9
Income $AUD/month		
< 500	6	28.6
501-1000	4	19.0
1001-1500	2	9.5
1501-2000	6	28.6
> 2000	3	14.3
Religion		
Buddhist	9	42.9
Taoism	7	33.3
Christian	2	9.5
Atheist	3	14.3
Obstetric data		
Parity		
Two	18	85.7
Three	3	14.3
Reasons for primary CS		
Prolonged labour	8	38.1
Foetal distress	3	14.3
Mal-presentation	4	19.1
Preeclampsia	2	9.5
Other	4	19.0

### Observation of consultation between obstetricians and pregnant women

Of the 21 pregnant women who participated in the study, only nine were observed during the consultation with their obstetrician. A total of 12 pregnant women were not observed because their obstetricians did not agree to participate in the study. Interactions between obstetricians and pregnant women were observed during consultations in the Outpatient Department of Obstetrics and Gynaecology, when women were 33-34 weeks’ gestation. Observational data were recorded and field notes were collected. During the consultation, women seldom asked questions. In most consultations obstetricians provided women with routine antenatal examinations such as checking the foetal heart beat and measuring the fundal height of the uterus. They did not provide any counselling regarding mode of birth. On average, each consultation was completed within 5-8 min.

### Pregnant women’s interviews

Ensuring the safety of the mother and baby was the overriding theme that emerged from the interviews with pregnant women. Two sub-themes within this overriding theme emerged: Influences on women’s decision making and decision-making processes of women. Across these sub-themes, six categories were developed from thirteen sub-categories (Table 2).

**Table 2:** Categories and themes synthesized to core theme, ensuring the safety of mother and baby

Core theme	Ensuring the safety of mother and baby	
Themes	Categories	Sub-categories
Influences on women’s decision-making	Previous birth experience	Positive experience Negatives experience
	Evaluation of mode of birth	Positive evaluation Negative evaluation
	Concern about the risks of vaginal birth	Uterine rupture Labour pain
	Current pregnancy situation	Foetal presentation Foetal size
	Informative resources	Obstetrician’s recommendations The experience of significant others Impact of internet
	Health insurance	National Health Insurance Private insurance
Decision-making processes of women	Searching for information Listening obstetricians’ professional judgment Evaluating alternatives Making a decision regarding mode of birth	

#### Ensuring the safety of mother and baby

Women were concerned about their health and their baby’s wellbeing. Most women in this study regarded the health and wellbeing of mothers and babies as the first consideration when making a decision regarding mode of birth. Two sub-themes were reflected in this theme: influences on women’s decision-making and the decision-making processes of women.

#### Influences on women’s decision-making

##### Previous birth experience

Women’s previous birth experience was the most frequently cited individual factor that affected women’s decisions. Often women who had a positive birth experience for their previous birth opted for the same mode of birth for the subsequent birth. Conversely, women selected a different mode of birth if they had negative experiences during the previous birth.

### Positive experience

Three women were confident in having a VBAC, based on their previous vaginal birth experience. Similarly, two women who had a positive experience of a CS selected RCS

*16-03: I had a vaginal birth for my first baby and a caesarean section for my second baby. The experience of vaginal birth was excellent, so I have been thinking about having a vaginal birth for my third baby. I prefer to have a vaginal birth.* (Vaginal birth)

*18-09: I feel that caesarean section was not really painful. I feel OK and I can endure it. Since I had the experience of a caesarean section for my first child, I will have my second child through the same method of caesarean section.* (RCS)

### Negative experiences

A total of eight women had experienced a previous CS due to an unsuccessful induction of labour and in these instances cervical dilatation was limited to less than 2 cm during latent stage or 7-8 cm during active stage. These women lacked confidence with VBAC for this birth because of the previously unsuccessful experience of induction of labour.

*05-01: Initially, I decided to have vaginal birth for the first birth but the doctor said it was less than two centimetres. So, I had induction of labour. However, the labour course was prolonged and I couldn’t give birth for a long time.. So in the end, I still had a caesarean section. I am afraid to experience the same situation as before. So I have decided to have a caesarean section this time.*

Two women who had a negative experience with a CS were willing to attempt a VBAC. These two women reported that they were reluctant to have RCS because of the severe back pain and fear of anaesthesia from their last birth experience.

*06-02: I felt that my caesarean section was terrible because it required anaesthesia and I felt terrible.*

#### Concern about the risks of vaginal birth

Concern about vaginal birth contributed to women selecting RCS. Two sub-categories were identified, uterine rupture and labour pain.

### Uterine rupture

Some women were concerned about the issue of uterine rupture. They were fearful of uterine rupture associated with vaginal birth. Concern about the risk of uterine rupture in relation to the interval between previous CS and this pregnancy and caesarean wound healing was reported by women who had a strong intention to attempt VBAC. These women reported they had at least over a three-year interval between this birth and the previous birth and they were more willing to attempt VBAC.

*11-02: Because I felt that [a caesarean section] was much safer, I was afraid that if the interval since the wound was too short, it might have caused problems at that time.*

### Labour pain

Women were also fearful of not having the ability to cope with labour and birth because of fear of pain. A total of five women were reluctant to attempt VBAC because they were concerned about failure of VBAC leading to experiences of pain. Of the five women, one woman did not attempt a VBAC and four women underwent VBAC for their previous birth that resulted in a RCS. Women were concerned they would experience the pain of a labour and, in the event a CS was required, wound pain from the operation would result.

*05-02: I am afraid that the result would be the same; that is, I will experience the pain twice!*

#### Evaluation of modes of birth

Women evaluated the different modes of birth and compared the advantages and disadvantages between CS and vaginal birth before deciding to have a RCS or a VBAC.

### Positive evaluation

The majority of women stated that a vaginal birth is better than CS for mothers’ and babies’ health. In this study, three women had experienced births involving both a CS and vaginal birth; they were all determined to attempt a VBAC for the forthcoming birth.

*07-01: It is for a quick recovery. My first baby was delivered by caesarean section, and my second baby was by vaginal birth. Comparing the two methods, I think the recovery was faster for the vaginal birth.*

Some women had a CS based on the consideration of safety and convenience. CS from their reports was viewed as much safer and more convenient than the uncertainty of a vaginal birth.

*15-18: I felt that this method was more convenient. You do not need to worry when there is sudden and unexpected pain in the abdomen. I love things that can be done in accordance with a plan.*

### Negative evaluation

Women compared the difference in pain and complications to evaluate the relative disadvantages between vaginal birth and CS. Pain was the most frequently cited negative influence on decision-making offered by women. They stated that vaginal birth was associated with pain before birth while there was pain after birth with CS.

*16-04: I approve of vaginal birth more, and because I had a caesarean section before, I want to have a vaginal birth even more [for my next birth]. Because a caesarean section means that you feel the pain afterwards, but for a vaginal birth, you feel the pain on the day of the birth, and it’s okay afterwards. For me, I am afraid of pain so I approve more of having a natural birth.*

When women compared the potential complications of the two modes of birth, they were concerned with the complication of adhesions if they had RCS, while incontinence was noted as the biggest concern by women if they selected VBAC.

*07-17:For my previous birth, it was for almost two years, and I still have that kind of incontinence problem. I think it had a very far reaching impact!*

#### Current pregnancy situation

The situation of the current pregnancy affected women’s decisions. In particular, if women wanted to have a VBAC, they were concerned about foetal presentation and foetal size.

### Foetal presentation

Women who intended to attempt a VBAC continued to accept ultrasound examination before 38 weeks’ gestation to confirm foetal presentation. Three women had foetal mal-presentation detected before 35 weeks’ gestation but they all turned to a cephalic presentation between 35 and 38 weeks’ gestation. All three women continued to plan for a RCS because they reported that they had already made this decision.

*15-25: It was about the thirty-second week. The prenatal examination revealed an abnormal foetal position. In the later period (35weeks), I thought that I should make an appointment to have a caesarean section at that time, even though the foetal position was normal.*

Foetal size.

Women were also concerned about foetal size. They were concerned that they may not have VBAC if the foetus was too big.

*17-19: Actually, my fear was more or less in my mind, and then based on the size of my baby, I was more worried. But I just wanted to see the baby’s condition because my baby was not big, and then I thought, okay! Let’s give it a try.*

#### Information resources

Women did not receive formal written information regarding VBAC from obstetricians and nurses. Instead, they received information from several sources, including obstetricians’ recommendations, the experience of significant others, and impact of internet.

### Obstetrician’s recommendations

Of the information sources, obstetricians’ recommendations were a critical factor influencing women’s decisions. Eight women who decided to have a RCS were influenced by their obstetricians’ recommendations that once a caesarean has been performed then the next birth should also be a caesarean**.** Four women reported that they did not receive any explanation from obstetricians but they complied with their obstetrician’s choice.

*13-01: Caesarean delivery! Doctor Z decided! It was not my decision. The doctor did not tell me the reason; he just took a look. Because my first baby was born with his assistance, he directly said caesarean delivery this time.*

### The experience of significant others

Some women made birth choices based on their significant others’ experience. Women’s family members, in particular their mothers, mothers-in-law, sisters and sisters-in-law who had birth experiences were reported by women to have more influence on their decisions than other female friends or colleagues. The majority of significant others judged mode of birth based on their previous birth choices. For instance, they recommended RCS because they had a VBAC resulting in a RCS.

*05-15: Because my older sister had the same results for her first baby, she suffered bad pain twice, exactly the same situation. She wanted to have a vaginal birth the first time, thinking that it is better for both the baby and the pregnant woman. But she tried to have a vaginal birth and got the same result. I made the decision because I asked my older sister.*

### Impact of internet

Platforms such as Yahoo, Facebook, and Google were a common source of information for woman and some obstetricians also recommended women search for relevant information on the internet. On the internet, some women who had a previous CS shared their experiences regarding birth choices. These women stated that if you had the first baby by CS, you should have a CS for your second baby because the obstetrician made this recommendation. In addition, pregnant women were afraid to have a VBAC because of the dramatic descriptions of uterine rupture prevalent on the internet.

*03-06: Many Internet rumours have been spread that the wound may rupture before the birth, and that the amniotic fluid will come out or the baby’s hair will come out. Many opinions like that saying that vaginal birth after caesarean section also puts the baby and the mother at a high risk.*

#### Health insurance

Two sub-subcategories were identified, including National Health Insurance and private insurance. In Taiwan, the National Health Insurance System is a social insurance program administered by the government [39]. National Health Insurance is compulsory social insurance, providing all citizens with equal access to medical services. Insured people need to pay premiums regularly and they receive full medical care. Notably, National Health Insurance offers financial coverage for a subsequent pregnancy as long as women have had a previous CS.

### National Health Insurance

National Health Insurance reimburses health care providers on a fee-for-service basis. For some women, the financial factor did not affect their decision for RCS because of the overriding considerations for safety.

*14-13: The doctor said that the National Health Insurance program would cover it. If it did not provide cover, it would be very expensive. However, even in that situation, I would still choose a caesarean delivery because of the consideration of safety.*

### Private insurance

In contrast to National Health Insurance, private insurance has strict criteria of financial coverage for RCS in Taiwan. Most private insurance does not cover RCS if women do not undergo a VBAC. Although a few women agreed to have a RCS based on their obstetricians’ recommendations for consideration of safety, they were still upset their private insurance could not cover the cost.

*19-30: I have private insurance! The doctor said that if you have an operation this time because of the previous caesarean delivery, it is not covered by insurance according to our insurance company.*

#### Decision-making processes of women

The decision-making process for women involved searching for information, listening to obstetricians’ professional judgment, evaluating alternatives, and making a decision regarding mode of birth.

##### Searching for information

Women sought relevant information regarding mode of birth from obstetricians, significant others or the internet and then discussed the options with obstetricians to confirm the mode of birth. Some (4/21) women did not receive information regarding VBAC from obstetricians and they complied with a RCS arrangement because their obstetrician had scheduled a RCS for them, without inquiring about the women’s intention. These women accepted a decision for RCS in the first trimester of pregnancy.

##### Listening to obstetricians’ professional judgment

Although most women in this study wished for as natural a birth as possible, a total of 13 women on hearing their obstetrician’s explanation and recommendation regarding mode of birth, selected RCS. The explanation of risks about uterine rupture from some obstetricians influenced women’s decisions. Some more experienced obstetricians explained the monitoring systems, such as cardiotocography, used during labour to detect complications in women with risk factors, while the potential for urine rupture was described by less experienced obstetricians. Women were reluctant to have VBAC if the potential for uterine rupture was described.

##### Evaluating alternatives

Women who had a preference for vaginal birth evaluated the advantages and disadvantages of alternatives, the condition of their health and that of their unborn baby, and obstetricians’ professional recommendations to decide whether they would have a VBAC. In particular, women considered the interval between their pregnancies, current pregnancy status (such as foetal presentation and the estimated foetal size). In contrast, women who were willing to have VBAC reported equivalent proportions of risks between VBAC and vaginal birth, over a five-year birth interval between the current and the previous birth, and/or a well-equipped medical centre to manage emergency situations. These women were confident with vaginal birth and viewed vaginal birth positively.

##### Making a decision regarding mode of birth

Some women made a decision for RCS/VBAC following evaluation of alternatives. A total of nine women who intended to have VBAC decided on this mode before 35-37 weeks gestation. Twelve women decided to have a RCS after considering their own condition and that of the foetus or once the risks were explained by obstetricians.

### Postnatal women’s interviews

Of the nine women who attempted a VBAC, six women were in their second pregnancy and three women were pregnant for the third time. All women who were in their third pregnancy achieved a VBAC; another two women achieved a vaginal birth; two women planned a VBAC but then scheduled a RCS because labour did not commence spontaneously by 38 weeks’ gestation; and two women attempted a VBAC that resulted in a RCS as their labour course progressed slowly. Of the twelve women planning to give birth by RCS, all women delivered their second baby with RCS (Table 3). Half of the 21 postnatal women reported that they were satisfied with their decisions regarding preferred mode of birth, while a third of women accepted the outcome because of its perceived safety. Postnatal women evaluated their decisions regarding mode of birth in three areas, reflection on birth choices, reflection on factors influencing decisions, and reflection on outcomes of decisions.

**Table 3:** Women’s intentions and their actual mode of birth

Birth mode	Intended mode of birth	Actual mode of birth	
		RCS	VBAC
VBAC	9 (42.9%)	4 (19.1%)	5 (23.8%)
RCS	12 (57.1%)	12 (57.1%)	0 (00.0%)
Total	21(100.0%)	16 (76.2%)	5 (23.8%)

#### Reflection on birth choice

Postnatal women who planned a VBAC reflected on the process of labour to form a view about the reason for their mode of birth. Women described being carefully monitored for signs of labour to evaluate whether the labour had started, such as a bloody show, water breaking, and labour pains.

*17-22: I had labour induction for eight hours. After nine hours, [the contractions] were getting faster. I was three centimetres in nearly nine hours, but afterwards, [they became] faster, and I was fully dilated in half an hour.* (VBAC.)

Postnatal women who had RCS reflected on the process of RCS to evaluate their operative birth. These women reflected on their experience of an operation for their primary caesarean delivery compared with the RCS.

*14-23: The doctor scheduled the operation for me and then performed a caesarean section directly. Last time, I did not feel anything; I was entirely asleep. But this time, I could feel pain and it felt quite terrible.* (RCS.)

#### Reflections on factors influencing decisions

After birth, postnatal women reflected on factors influencing their birth decisions. Obstetrician’s advice was the main factor influencing a woman’s decision. Some women in the study stated their obstetricians did not provide them with any information to help them make birth choices but they complied with obstetricians’ recommendation to have RCS. They also expressed their fear about asking questions of their obstetrician because the obstetrician had many patients waiting for consultation.

*19-47 The doctor! I was that kind of person! What the doctor told me, that method, I just followed what he said.* (RCS.)

*10-47 The doctor was too busy. When you asked him questions, he just briefly understated it, telling you in just a few words. He could not answer all your doubts.* (RCS.)

The study hospital offered information regarding general antenatal and postnatal care only. One woman, who attempted a VBAC that resulted in a RCS, attributed her failure to poor information provision.

*08-58: I felt that the information was insufficient!* (Woman attempted a VBAC that resulted in a RCS.)

#### Refection on outcomes of decisions

Postnatal women reflected on the outcomes of their decisions regarding mode of birth. All postnatal women were happy with their baby’s health and wellbeing after birth except for one woman who attempted a VBAC that resulted in a RCS. These women reflected on their decisions from the perspectives of their recovery and their baby’s health at birth.

*02-39: I still felt weak but it was much better [than after the previous birth]. This birth was much better than the previous birth.* (*Women had a* RCS.)

*17-03: My physical strength was really different! Body recovery was pretty good. One month after birth, I felt good. My physical strength and vigour were good.* (*Women had* a VBAC.)*.*

## Discussion

A qualitative approach was used to elicit Taiwanese women’s decision-making processes and the influences on their mode of birth following a previous CS. Women’s decision-making was identified using multiple data collection methods, including in-depth interviews, observations and field notes. In particular, this study used in-depth interviews to capture individual participants’ viewpoints.

**Fig. 2:**
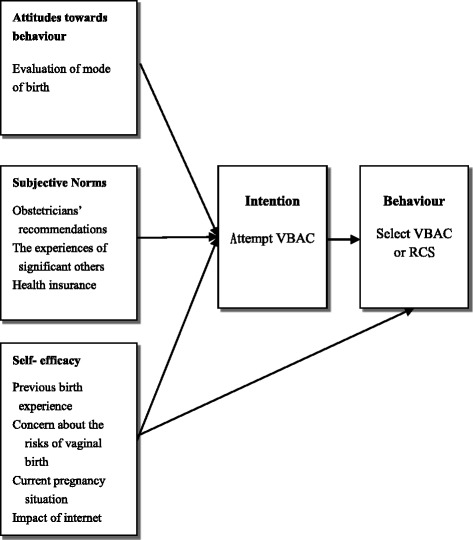
Taiwanese women’s decision-making regarding mode of birth as applied to TPB

The Theory of Planned Behaviour (TPB) was used to assist in the interpretation of the findings. Specifically, the TPB framework assisted in discerning the reasons underpinning women’s decisions and also assisted to understand women’s actions regarding mode of birth. The TPB includes three psychometric determinants influencing human behavioural intentions [40]. These determinants are attitudes towards a behaviour, subjective norms and perceived behavioural control [41]. Taiwanese women’s decisions were influenced by internal and external factors. Internal or personal factors related to an evaluation of mode of birth were reflected in the ‘attitudes toward behaviour’ construct of the TPB (Fig. 2). In this study, women who evaluated vaginal birth in a positive light were more willing to attempt VBAC. Conversely, women who had a negative attitude towards VBAC selected RCS. This finding is consistent with previous studies [14, 42].

Subjective norms include external factors influencing women’s decision making such as obstetricians’ recommendations, the experiences of significant others and health insurance. In this study, obstetricians’ recommendations and the experiences of significant others (female families or friends) played an important role in women’s decision-making. Obstetricians have been described as a major factor in driving the CS rate upward [10]. Research has shown that hospital physicians were the most frequent information providers; however, they provided women with information about procedural issues rather than possible health risks and benefits [43, 44]. In the present study, unbalanced explanations regarding the risk of uterine rupture potentially affected women in making decisions in favour of RCS. This finding corroborates findings of a recent Australian study [45]. Australian women who chose RCS reported that VBAC was the most risky option, based on the information they received from hospital obstetricians [45]. In addition, some pregnant women in the study complied with obstetricians’ recommendation to opt for RCS because they found it difficult to oppose obstetricians’ opinions. Medical recommendations, especially during the birth, were experienced as powerful and difficult for women to oppose [45]. Power and a knowledge imbalance, as well as trusting relationships with obstetricians continue to reinforce this momentum. Positive benefits of midwife led care indicate the recommencement of midwifery practice in Taiwan to optimise maternal care [46–49].

Perceived behavioural control is related to women’s intention to participate in decision-making. Self-efficacy is a key concept of perceived behavioural control [40]. Four constructs in our findings are related to women’s self-efficacy, including previous birth experience, current pregnancy situation, concern about the risks of vaginal birth and impact of the internet. In this study, women who had a high sense of self-efficacy were more willing to attempt VBAC. Several studies have demonstrated that previous experiences influenced decision-making in women who had had a previous CS and these experiences have been found to affect women’s subsequent birth choice [16, 50–53]. In the present study, Taiwanese women who had a positive experience selected the same mode of birth as their previous birth, while women who had a negative experience chose a different mode of birth. These findings are similar to those of a recent study, in which 85% of women who had an RCS stated they would make the same choice again, compared to 70.1% for the planned VBAC group [53]. In this study, most women lacked confidence to have VBAC because of their previous negative experience of induction. This finding is supported by findings of another Taiwanese study [54]. According to Kuan [54], Taiwanese women who were aiming to birth vaginally but had to have a CS were aware of medical intervention practices and they requested CS out of fear of “suffering twice”. Kuan argued that Taiwanese women requested a CS for their first birth because hospitals enforce a significant amount of medical intervention [54]. Because of concern about the risks of vaginal birth, some women considered RCS as the safest method for mother and baby based on medical perspectives; they were therefore unwilling to attempt VBAC. However, this assumption has been based on a misunderstanding of the risks and benefits of VBAC [55]. In fact, several previous studies have shown that women who have had a previous CS lacked knowledge about the benefits and risks regarding the various birth options [12, 56, 57]. Internet has been found to play an important role for women in searching for information regarding mode of birth [44, 58]. Due to a lack of leaflets, booklets and newsletters regarding VBAC in organisations, Taiwanese women sought help from other women on the internet. However, a lack of comprehensive information and inaccurate information on the internet contributed to study participants’ decision to avoid an attempt of VBAC. The findings of the present study in relation to use of the internet for information were consistent with those of a recent Swedish study [58]. This highlights the need for high quality information provision if the misconceptions are to be corrected.

## Strengths and limitations

For geographical convenience, data were collected in a large private medical centre rather than in a public hospital or a clinic in Taiwan. Therefore, the findings may not be transferable to all pregnant women in Taiwan. Women were interviewed in a waiting room while awaiting their consultation, the location and time constraints may have limited their ability to fully share their stories. In spite of these limitations, the findings that emerged from participants’ interviews were meaningful and offered greater depth than a survey would have produced. Additionally, this study comprised of interviews with prenatal and postnatal women which was helpful in capturing their actual thinking regarding mode of birth from different perspectives. Observation of women-obstetrician dyads during consultations enabled examination of the actual interactions between obstetricians and pregnant women, thereby providing a deeper understanding of the information sharing and decision-making process than would not have been obtained through the individual interviews alone.

## Conclusions

The health and wellbeing of mother and baby were the major concern for women deciding on a mode of birth after a previous CS. Influenced by internal and external factors, women’s interactions with obstetricians regarding mode of birth choices varied from complying with obstetricians’ recommendations to shared communication styles. In the present study, if women participated passively in the decision-making process regarding their birth choices, their choices were primarily guided by risk reduction of uterine rupture. They were particularly influenced by obstetricians’ recommendations in early pregnancy. By contrast, women who actively participated in decision-making regarding their birth choices were guided by a previous experience of vaginal birth. These women discussed with obstetricians their intention to attempt VBAC and were actively involved in the decision-making.

All women have the right to be informed of alternative options of birth. Internal factors, in particular, establishing a supportive birth environment such as Next Birth after Caesarean (NBAC) clinics is helpful in facilitating women’s confidence in VBAC. Routine provision of explanations by obstetricians regarding risks associated with alternative birth options, in addition to financial coverage for RCS from National Health Insurance, would assist women’s decision-making. Establishment of a website of decision aids to provide women with reliable information about birthing options may also assist women’s decision-making. Finally, midwife-led models of care for information provision may hold promise for reducing risks of birth in Taiwan.

## Additional file

Additional file 1: Interview guide. (DOCX 16 kb)

## Abbreviations

CS: Caesarean Section
NBAC: Next Birth after Caesarean
RCS: Repeat Caesarean Section
VBAC: Vaginal Birth after Caesarean
