# Supplementary material for: Women’s decision-making processes and the influences on their mode of birth following a previous caesarean section in Taiwan: a qualitative study
Source: BMC Pregnancy Childbirth. 2018 Jan 17;18:31. doi: 10.1186/s12884-018-1661-0 (PMC5773050; doi:10.1186/s12884-018-1661-0)
Supplement: Additional file 1: — Interview guide. (DOCX 16 kb) [file 12884_2018_1661_MOESM1_ESM.docx]

**Interview guide for women**

***Prenatal interview for pregnant women who have had a previous CS***

1. Please tell me your plan regarding mode of birth?
2. How did you make this decision regarding mode of birth?
3. Did your obstetrician assist you to make this birth choice? In what way?
4. How did you acquire information about your mode of birth?
5. What is your family’s attitude toward mode of delivery?
6. What other factors influenced your decision? Cultural considerations? Financial constraints? Health insurance.......

***Postnatal women who have had a previous CS***

1. Please tell me what influenced your plan for mode of birth?
2. Did your birth happen to according to your plan? If not, were you involved in the decision about the change of plan?
3. Please describe counselling by health professionals about VBAC or RCS?
4. How do think obstetricians’ roles make an impact during the decision-making process?
5. How do you see your role in this decision-making process?
6. What information did you need to assist you to decide on mode of birth?
7. In hindsight, are you satisfied with your decision regarding mode of birth?
